# Exploring the Cultivable *Ectocarpus* Microbiome

**DOI:** 10.3389/fmicb.2017.02456

**Published:** 2017-12-11

**Authors:** Hetty KleinJan, Christian Jeanthon, Catherine Boyen, Simon M. Dittami

**Affiliations:** ^1^Sorbonne Universités, CNRS-UPMC, Station Biologique de Roscoff, UMR8227, Integrative Biology of Marine Models, Roscoff, France; ^2^CNRS, Station Biologique de Roscoff, UMR7144, Adaptation et Diversité en Milieu Marin, Roscoff, France; ^3^Sorbonne Universités, UPMC Univ Paris 06, Station Biologique de Roscoff, UMR7144, Adaptation et Diversité en Milieu Marin, Roscoff, France

**Keywords:** *Ectocarpus*, holobiont, bacterial cultivation, brown macroalgae, dilution-to-extinction, metabarcoding

## Abstract

Coastal areas form the major habitat of brown macroalgae, photosynthetic multicellular eukaryotes that have great ecological value and industrial potential. Macroalgal growth, development, and physiology are influenced by the microbial community they accommodate. Studying the algal microbiome should thus increase our fundamental understanding of algal biology and may help to improve culturing efforts. Currently, a freshwater strain of the brown macroalga *Ectocarpus subulatus* is being developed as a model organism for brown macroalgal physiology and algal microbiome studies. It can grow in high and low salinities depending on which microbes it hosts. However, the molecular mechanisms involved in this process are still unclear. Cultivation of *Ectocarpus*-associated bacteria is the first step toward the development of a model system for *in vitro* functional studies of brown macroalgal–bacterial interactions during abiotic stress. The main aim of the present study is thus to provide an extensive collection of cultivable *E*. *subulatus*-associated bacteria. To meet the variety of metabolic demands of *Ectocarpus*-associated bacteria, several isolation techniques were applied, i.e., direct plating and dilution-to-extinction cultivation techniques, each with chemically defined and undefined bacterial growth media. Algal tissue and algal growth media were directly used as inoculum, or they were pretreated with antibiotics, by filtration, or by digestion of algal cell walls. In total, 388 isolates were identified falling into 33 genera (46 distinct strains), of which *Halomonas* (*Gammaproteobacteria*), *Bosea* (*Alphaproteobacteria*), and *Limnobacter* (*Betaproteobacteria*) were the most abundant. Comparisons with 16S rRNA gene metabarcoding data showed that culturability in this study was remarkably high (∼50%), although several cultivable strains were not detected or only present in extremely low abundance in the libraries. These undetected bacteria could be considered as part of the rare biosphere and they may form the basis for the temporal changes in the *Ectocarpus* microbiome.

## Introduction

Coastal areas form the major habitat of brown macroalgae, photosynthetic eukaryotic organisms that are important primary producers and form biodiversity hotspots for other marine (macro)organisms by providing them with food and shelter (Thornber et al., 2016). The seaweed surface is a highly attractive substrate for the settlement of marine microorganisms, due to the fact that they actively excrete carbohydrates and other organic or growth-promoting substances (Salaün et al., 2012; Goecke et al., 2013b) that can be rapidly utilized by bacteria. Several stable relationships exist that have been shown to benefit brown macroalgal hosts (Goecke et al., 2010; Hollants et al., 2013; Singh and Reddy, 2016). Algae-associated (symbiotic) microbes can, for example, communicate on a chemical level through the provision of growth hormones (Pedersén, 1973), vitamins (Pedersén, 1969; Croft et al., 2005), or morphogens (Tapia et al., 2016), and some algal–bacterial interactions are known to affect biofouling and pathogenic invasion by other microorganisms (Singh and Reddy, 2014). Due to the tight relationships and functional co-dependencies between algae and their associated microbiomes, both can be seen as one functional entity or “holobiont" (Zilber-Rosenberg and Rosenberg, 2008; Egan et al., 2013).

Elucidating the functions and molecular mechanisms that shape the algal holobiont is of crucial importance, not only for the fundamental understanding of macroalgal functioning in marine ecosystems, but also to improve macroalgal culturing, an industry that has increased intensively over the last decade due to the growing interest in algae as a source for nutrients, chemicals, and bioactive compounds (Wells et al., 2017). *In vitro* studies of the commercially valuable and environmentally most relevant brown macroalgae (kelps, order *Laminariales*) remain challenging due to their size and complex life cycles (Peters et al., 2004). Model organisms, such as the filamentous brown alga *Ectocarpus* are therefore an essential tool to enable functional studies on algal–bacterial interactions in the laboratory. *Ectocarpus* is easily cultivable *in vitro*, has a short life cycle and a relatively small genome which has been sequenced several years ago (Peters et al., 2004; Cock et al., 2010).

Here, we study the microbiome of a freshwater strain of *Ectocarpus subulatus* (West and Kraft, 1996). The transition to fresh water is a rare event in brown algae that occurred in only a few species (Dittami et al., 2017). The examined strain is currently the only publicly available freshwater isolate within the Ectocarpales, and it is still able to grow in both seawater and freshwater (Dittami et al., 2012). This and other isolates of the same species are known for their particularly high tolerance to abiotic stressors (Bolton, 1983; Peters et al., 2015) and are being developed as a model to study brown algal adaptation and acclimation. These processes, and in particular algal growth in fresh water, have been shown to depend on interactions with symbiotic bacteria (Dittami et al., 2016).

The aim of the present study is to develop an extensive collection of cultivable *E*. *subulatus*-associated bacteria that can be used to study the functions of bacterial symbionts during abiotic stress in controllable and reproducible experimental settings, using the freshwater strain of *E. subulatus* as a model. Different bacterial isolation techniques were applied in parallel to increase the number and diversity of cultivable strains, i.e., direct plating and dilution-to-extinction cultivation techniques, each with chemically defined and undefined bacterial growth media. Algal tissue and algal growth media were directly used as inoculum, or they were pretreated with antibiotics, by filtration, or by digestion of algal cell walls. Our data show an overall high culturability of *Ectocarpus*-associated bacteria including a high number of low abundance taxa.

## Materials and Methods

### Cultivation of Algae – Starting Material for Isolation of Bacterial Symbionts

All experiments were carried out using sporophytes of the *E. subulatus* freshwater strain (EC371, accession CCAP 1310/196, West and Kraft, 1996). This culture was obtained from Bezhin Rosko (Santec, France) in 2007 and maintained in our laboratory under the following conditions since then: cultures of EC371 were grown in Petri dishes (90 mm Ø) in natural seawater (NSW; collected in Roscoff 48°46′40′′ N, 3°56′15′′ W, 0.45 μm filtered, autoclaved at 120°C for 20 min), or in diluted seawater-based medium (DNSW; by 20-fold dilution of natural seawater with distilled water). Both media were enriched with Provasoli nutrients (Starr and Zeikus, 1993) and cultures kept at 13°C with a 12 h dark-light cycle (photon flux density 20 μmol m^-2^ s^-1^).

### Isolation and Characterization of Algae-Associated Bacteria

**FIGURE 1:**
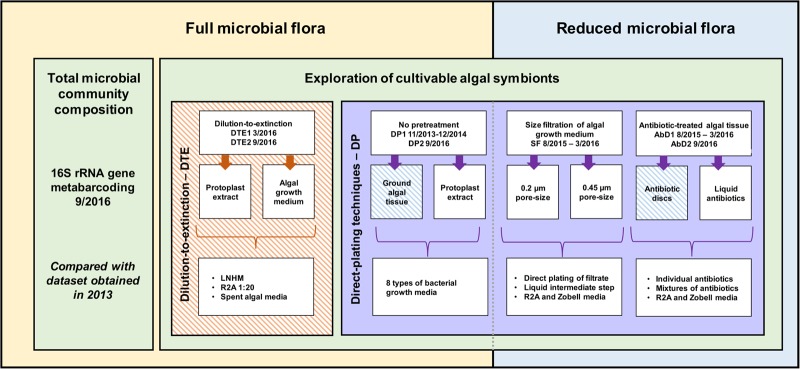
Overview of the methodology and cultivation strategies used to cultivate algae-associated bacteria. On one hand, direct inoculation with algal tissue and/or algal growth medium was used (yellow), while on the other hand, the microbial community was reduced before inoculation (blue). Additionally, a distinction can be made between direct plating (DP, purple) with and without pretreatment, and dilution-to-extinction cultivation (orange). 16S rRNA gene metabarcoding of the total prokaryotic community was carried out in parallel. Striped boxes indicate experiments that have been repeated twice within a 6 months interval for DTE1–DTE2, a 1-year interval for AbD1–AbD2, and a 3-year interval for DP1–DP2 and META13–META16 (Dittami et al., 2016).

A range of cultivation strategies as well as bacterial growth media was exploited. The starting material for bacterial isolation was EC371 grown with its full microbial flora (direct plating and dilution-to-extinction cultivation) and EC371 with a reduced microbial flora (size-fractionation and antibiotic treatment), both originating from the same algal culture. Algal subcultures were sampled 5–10 days after the last change in medium. Algal growth medium and ground algal tissue, in NSW and DNSW were used. The isolation experiments took place from November 2013 to September 2016. Three selected cultivation experiments (dilution-to-extinction cultivation, direct plating with antibiotics; direct plating without pretreatment) were repeated under identical conditions after 6 months, 1 year, or 3 years, respectively, to assess reproducibility of the results over time. An overview of the isolation methods and cultivation strategies is provided in **Figure 1**.

#### Isolation of Bacteria from Algae with Their Full Microbial Flora

##### Direct plating techniques

To isolate bacteria, algal growth media, ground algal tissue, and algal protoplast digest product of EC371 grown in DNSW or NSW were directly plated (DP) on eight different growth media solidified with 1.5% agar. The eight bacterial growth media were: R2A prepared in distilled water (adapted from Reasoner and Geldreich, 1985); R2A prepared in natural seawater instead of distilled water; Zobell marine agar (Zobell, 1941); Zobell marine agar with 16-fold reduced salinity; *Ectocarpus*-based medium (ground *E. subulatus* 5 g DW⋅L^-1^; Peptone 0.5 g L^-1^, Provasoli nutrients 10 ml L^-1^, 5% NSW); Peptone Yeast Glucose (PYG) agar (Peptone 0.5 g L^-1^; Yeast Extract 0.5 g L^-1^; Glucose 0.5 g L^-1^); PYG with glucose replaced by mannitol (5 g L^-1^) and Provasoli nutrients 10 ml L^-1^; and LB with NaCl (2 g L^-1^). In some cases, a liquid intermediate step was applied, and in all cases non-inoculated media and plates were included as negative controls. The exact recipes of the media can be found in **Supplementary Table S1** and the detailed experimental treatments in **Supplementary Table S3**. The algal protoplast digest product (used in dilution-to-extinction cultivation as well) was produced using the protocol from Coelho et al. (2012) with an additional 2.0 μm size-filtration after complete cell wall digestion (step 5) and the filtrate was used for direct plating. After incubation for up to 45 days at either 4, 13, 30°C, or room temperature (RT), 1–10 single colonies were picked randomly. Furthermore, any colonies that differed with regard to their shape, size or color were also included. The colonies were grown in liquid growth media and identified by sequencing their 16S rRNA gene via Sanger sequencing (as described below). Direct plating of ground EC371 tissue grown in NSW was repeated after 3 years. Due to the variety of experiments colony counts were variable, ranging between one and several 100 per plate.

##### Dilution-to-extinction cultivation

As a strategy to reduce nutrient competition between the cultivable members of the EC371 microbiome, the high throughput dilution-to-extinction (DTE) cultivation approach was used as originally described by Connon and Giovannoni (2002): microbial communities from either algal growth medium or algal protoplast digest product (see the previous section) were 0.6 μm-filtered to remove microbial and carbohydrate aggregates, diluted to a predefined cell number, and distributed into 96-well deep well plates with low-nutrient media. Algal tissue may harbor cell-wall attached bacteria whose numbers cannot be determined with flow cytometry. In addition, the algal fragments block the flow cytometer which also prevents correct cell counting. Therefore, algal tissue could not be directly used in dilution-to-extinction experiments. Four liquid bacterial growth media were used to cultivate bacteria: 20-fold diluted R2A prepared in DNSW with starch replaced by alginate (0.025 g L^-1^); Low Nutrient Heterotrophic Medium (LNHM) with 0.001 g L^-1^ mannitol (adapted from Cho and Giovannoni, 2004; Stingl et al., 2007, 2008; Carini et al., 2012; Jimenez-Infante et al., 2014) and 2 and 7 weeks old spent EC371 growth medium (5% NSW). Recipes can be found in **Supplementary Table S1**. For R2A and Zobell media, stock solutions of the individual components were prepared and autoclaved separately and the final bacterial growth medium was prepared on the day of the experiment. For LNHM, stock solutions were 0.2 μm filter-sterilized but not autoclaved. On the day of the experiment, individual components were mixed, the pH was adjusted to 7.3, and the bacterial growth media was filter-sterilized (0.1 μm) and divided into 96-well deep well plates before inoculation (0.5 ml/well). Preliminary tests of inoculations with 3, 1, and 0.5 cells/well, showed that 0.5 cells/well was the optimal inoculation density to limit the occurrence of bacterial mixtures and to obtain pure bacterial clones. Non-inoculated bacterial growth medium was used as a negative control. The experiment was performed twice within a 6 month interval (DTE1 in March 2016 and DTE2 in September 2016). Flow cytometry was used to obtain both the bacterial cell counts of the inocula and to monitor bacterial growth. After 4 weeks of incubation (16°C, 12:12 h dark:light cycle, 27 μmol s^-1^ m^-2^), bacterial growth was screened by flow cytometry using a BD Accuri C6 cytometer (BD Biosciences): 100 μl of the cultures were fixed with glutaraldehyde (0.25%, final concentration) and stained with Sybr Green (Life Technologies) as described by Marie et al. (1997). Wells with cell densities of 10^4^ cells/ml and higher were considered positive. The number of cultivable bacteria n_cult_ in the original inoculum was estimated based on the proportion of negative wells (p_neg_) according to a Poisson distribution using the formula n_cult_ = ln(1/p_neg_)⋅w, where w is the total number of wells inoculated (Button et al., 1993). This allowed for the calculation of the ratio of cultivable to total bacteria (the latter determined via flow cytometry) in these experiments (estimated culturability).

#### Isolation of Bacteria from Algae with a Reduced Microbial Flora

##### Size-fractionation of algal growth media

As a second strategy to reduce bacterial cell numbers before plating, size-fractionation (SF) was used to facilitate the growth of smaller and less abundant bacterial strains. EC371 culture medium was filtered with 0.2 (SF0.2), 0.45 (SF0.45), or 40 (SF40) μm pore-size, and 50 μl filtrate were directly plated on R2A or Zobell agar. At the same time, 100 μl filtrate were used to inoculate liquid R2A or Zobell as an intermediate to enhance bacterial growth before plating. After 5–8 days of incubation at RT, 50 μl of the liquid culture were plated on solidified R2A and/or Zobell. In both cases, plates were incubated until single colonies were visible (3–20 days) and the latter identified with 16S rRNA amplicon sequencing as described below.

##### Antibiotic-treatments of algal tissue and growth media

Antibiotics were used to reduce the abundance of dominant bacterial strains from the algal tissue and/or growth media, in our case especially *Halomonas* sp., and to facilitate the growth of other less abundant or slower-growing bacteria. Algal growth media and/or ground algal tissue was spread on R2A agar plates and incubated with two antibiotic disks (AbD1 and AbD2; Antimicrobial Susceptibility Disks, Bio-Rad Laboratories, Inc., France) for 5 days at RT, using antibiotics that were shown to be effective against *Ectocarpus*-derived *Halomonas*. Alternatively, algal subcultures of the same strain were treated with liquid antibiotics (AbL) for 3 days before plating on R2A or Zobell, whereafter 50 μl were plated on solidified R2A or Zobell. An overview of the antibiotics (disks and liquid) and their concentration can be found in **Supplementary Table S2**. Plates were incubated (3–20 days) until single colonies were visible and the latter identified with 16S rRNA amplicon sequencing as described below. Two experiments using antibiotic-treated algal tissue (AbD2 with chloramphenicol and erythromycin) were repeated after 1 year.

#### Bacterial Identification by 16S rRNA Gene Sequencing

To identify bacterial isolates, single colonies were grown in the corresponding liquid growth media until maximal density was reached. Approximately 50–100 μl of cultures were heated for 15 min at 95°C. Then the 16S rRNA gene was amplified using universal primers (8F 5′ AGAGTTTGATCCTGGCTCAG and 1492R 5′ GGTTACCTTGTTACGACTT from Weisburg et al., 1991) and the GoTaq polymerase in a PCR reaction with the following amplification conditions: 2 min 95°C; [1 min 95°C; 30 s 53°C; 3 min 72°C] 30 cycles; 5 min 72°C. In some cases a commercial kit was used to extract DNA (NucleoSpin^®^ Tissue, Machery-Nagel; support protocol for bacteria). The PCR products were purified using ExoSAP (Affymetrix, Inc., Thermo Fisher Scientific) and sequenced with Sanger technology (BigDye Xterminator v3.1 cycle sequencing kit, Applied Biosystems^®^, Thermo Fisher Scientific). Only the forward primer 8F was used for the sequencing reaction. For classification and analyses of the sequences, RDP classifier (Wang et al., 2007) and BLAST^1^ against the NCBI nr and 16S rRNA gene databases were used and sequences classified at the genus level if possible. Sequences were aligned^2^ and checked manually to verify mismatches and to identify distinct strains within a genus (>99% identity). The 16S rRNA gene sequences from each distinct cultivable strain were aligned using MAFFT version 7 (Katoh et al., 2002) and the G-INS-i algorithm. Only well-aligned positions with less than 5% alignment gaps (492 positions) were retained. Phylogenetic trees were reconstructed using the Maximum-Likelihood method implemented in MEGA6.06 (Tamura et al., 2013), and the GTR+G+I model. Five hundred bootstrap replicates were tested to assess the robustness of the tree. The unique 16S rRNA gene sequences were submitted to the European Molecular Biology Laboratory (EMBL) database and are available under project accession number PRJEB22665. Stocks of bacterial isolates were preserved in 40% glycerol at -80°C.

The numbers of sequences obtained per taxa were normalized against the total number of sequences obtained within the complete cultivation study. To assess cultivation biases statistically, the absolute abundances of cultivable isolates were analyzed in R (RStudio Team, 2016) with the Fisher-exact test and Bonferroni *post hoc* correction for multiple testing (α = 0.05, significant if *p* < 0.0011).

### 16S rRNA Gene Metabarcoding

To estimate the proportion of cultivable bacteria in our algal cultures, the collection of bacterial isolates was compared with 16S rRNA gene libraries from the same algal culture used for our isolation experiments. These libraries served as a reference to assess the total microbiome, including cultivated and non-cultivated bacteria; they were not used to infer diversity *per se*. EC371 cultures were grown for 9 weeks in seawater-based culture medium (changed on a monthly basis, last 1 week prior to sampling). Algal tissue was filtered with sterile coffee filters, dried on a paper-towel, and snap-frozen in liquid nitrogen. Four technical replicates were pooled two by two. Total DNA was isolated (NucleoSpin^®^ Plant II, Machery-Nagel; standard protocol) and purified with Clontech CHROMA SPIN^TM^-1000+DEPC-H2O Columns. The V3–V4 region of the 16S rRNA gene was amplified and sequenced with Illumina MiSeq technology by MWG Eurofins Biotech (Ebersberg, Germany) using their proprietary protocol. The first preliminary quality control was done with FastQC^3^, and fastq_quality_trimmer from the FASTX Toolkit^4^ was used to quality-trim and filter the 568,100 reads (quality threshold 25; minimum read length 200). The resulting 553,896 sequences (2.5% removed) were analyzed with Mothur (V.1.38.0) according to the MiSeq Standard Operating Procedures^5^ (Kozich et al., 2013). Filtered reads were assembled into 270,522 contigs, preclustered (allowing for four mismatches), and aligned with the non-redundant Silva SSU reference database version 123 (Quast et al., 2013). Chimeric sequences were removed using the Uchime algorithm (Edgar et al., 2011) implemented in Mothur, and the remaining sequences classified taxonomically using the method of Wang et al. (2007). Non-bacterial sequences were removed. The sequences were then clustered into operational taxonomic units (OTUs) at a 97% identity level and each OTU was classified taxonomically. All OTUs with *n* ≤ 5 sequences were removed (0.02%) resulting in a final data matrix with 217,923 sequences. The sequences obtained from cultivable isolates were compared with the 16S rRNA gene metabarcoding data using BLASTn searches against raw reads (99% identity) and consensus OTU sequences (97% identity). In addition, the current dataset (META2016-NSW) was compared to previous datasets (META2013-NSW, META2013-DNSW) obtained from the same algal strain 3 years earlier (Dittami et al., 2016). All counts for each individual OTU were normalized against the total number of sequences in the corresponding dataset. Raw Illumina reads were deposited at the European Nucleotide Archive under project accession number PRJEB22665. To compare the cultivable sequences and their abundance in the 16S metabarcoding data (META13-NSW and META16-NSW) a heatmap was created using the iTOL web application^6^ (Letunic and Bork, 2016). Log(x+1)-transformed data was used for OTU sequence counts and cultivation abundances. All datasets (three for metabarcoding, 10 for cultivation) were grouped by hierarchical clustering using Euclidean distance calculations and the average linkage method implemented in the pvclust R-package^7^ (Suzuki and Shimodaira, 2006). The resulting tree was tested using bootstrap analysis (500 replications). OTUs that did not correspond to cultivable strains are not shown in the graphical representation. In this manuscript, “isolate” refers to every bacterial culture for which a 16S rRNA sequence was obtained. All isolates with identical 16S rRNA sequences are considered to belong to the same “strain”.

## Results

### Isolation and Characterization of Algae-Associated Bacteria

#### Global Taxonomic Distribution of Cultivable Bacteria

**FIGURE 2:**
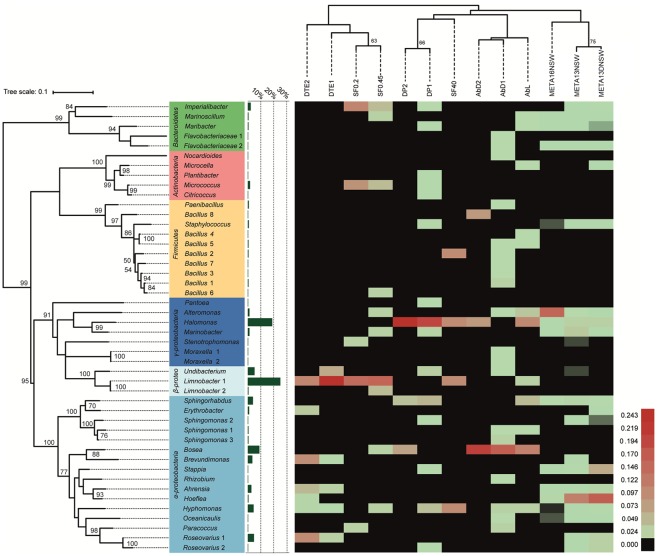
Heat-map of cultivation and metabarcoding data. The number of sequences was normalized and log(x+1)-transformed for each unique cultivable strain and each experimental treatment (DP, direct plating without pretreatment; AbD, DP with pretreatment with antibiotic disks; AbL, DP with pretreatment with liquid antibiotics; SF, DP with pretreatment by size-fractionation; DTE, dilution-to-extinction cultivation). A comparison is made with molecular data from 16S rRNA metabarcoding (META16-NSW = this study; META13-NSW and META13-DNSW = previous study by Dittami et al., 2016; uncultured OTUs not shown). Red colors indicate high abundance, while green corresponds to relatively low abundance. Black color indicates taxa/strains that were not retrieved/isolated. Experimental treatments are grouped (top dendogram) using hierarchical clustering (Euclidean distance, average linkage method) and the phylogenetic tree (left) was calculated using the Maximum-Likelihood method and the GTR+G+I model. Bootstrap analysis for both trees was done using 500 replications. Only bootstrap values ≥ 50 are shown. The bar graph (green) shows the proportion of isolates per unique strain obtained in the whole cultivation dataset.

16S rRNA gene sequences were obtained for 388 bacterial isolates and they were distributed among four phyla, 15 bacterial orders, 34 genera, and 46 taxonomically unique strains. Five genera encompassed more than one distinct strain (i.e., at least one verified mismatch in the 16S rRNA sequence): *Limnobacter* sp. (2), *Moraxella* sp. (2), *Sphingomonas* sp. (3), *Bacillus* sp. (8), and *Roseovarius* sp. (2). The most abundant phylum among the cultivable isolates was *Proteobacteria*, with 89% of all isolates and 26 unique strains belonging to this group. Within *Proteobacteria*, *Alpha*- and *Betaproteobacteria* accounted for 34 and 32% of the isolates, respectively. However, *Betaproteobacteria* comprised three unique strains, while *Alphaproteobacteria* comprised 16 unique strains in our experiments. 23% of proteobacterial isolates belonged to *Gammaproteobacteria*, covering seven unique strains. *Bacteroidetes* (4% of isolates), *Firmicutes* (4%), and *Actinobacteria* (3%) were cultivated less frequently compared to *Proteobacteria*. Despite their lower abundance, the three groups contribute considerably to the cultivable diversity, accounting for 20 out of 46 unique strains. The most abundant cultivable bacterial genera were *Limnobacter* (27% of all isolates), *Halomonas* (20%), and *Bosea* (9%). 80% of *Limnobacter* isolates were obtained from dilution-to-extinction cultivation experiments. *Halomonas* strains were predominantly cultivated using direct plating techniques and algae with full flora (84% of *Halomonas* isolates). For *Bosea*, most isolates (83%) originated from antibiotic-treated algae. An overview of all bacterial isolates characterized and their corresponding sequence abundances can be found in **Figure 2** and **Supplementary Table S3**.

#### Isolation of Bacteria from Algae with Their Full Microbial Flora

##### Direct plating of ground algae and algal protoplast extract (DP)

Direct plating of ground algal tissue and protoplast digest resulted in the isolation and characterization of 110 isolates corresponding to 17 strains of which seven were uniquely isolated with this method. The most frequently isolated strain was *Halomonas* sp., a gammaproteobacterium that makes up for 58% of isolates obtained with this method. Isolates of this strain originated predominantly from ground algal tissue rather than algal growth medium (*p* = 1.92E-13). After *Halomonas*, *Sphingopyxis* (10%), and *Hyphomonas* (8%) were the most frequently isolated taxa. Four isolates originated from protoplast extracts: *Imperialibacter* sp. (two isolates), *Sphingomonas* sp. (one isolate), and *Plantibacter* sp. (one isolate). Direct plating of algal tissue was repeated after 3 years and *Halomonas* sp. was again the most frequently isolated strain (9 out of 12 isolates). *Sphingomonas* 2, and *Plantibacter* sp. were two protoplast-specific strains obtained using DP, but they were only isolated once in the experiment.

##### Dilution-to-extinction cultivation (DTE)

One hundred and fifty isolates were identified and 16S rRNA gene sequences revealed eight unique strains. There were no isolates that were specific for the origin of the starting material used (protoplast extract or spent algal growth medium). The most abundant isolates belonged to the genus *Limnobacter* (55% of isolates), suggesting that they were the most abundant cultivable bacterium in the original algal cultures. Furthermore, five unique strains (*Brevundimonas*, *Erythrobacter*, *Hoeflea*, *Ahrensia*, and *Roseovarius 1*) were exclusively found with dilution-to-extinction cultivation. *Brevundimonas* sp. was significantly more isolated from algal growth media (*p* = 0.00045) compared to algal tissue/protoplast extract (**Supplementary Table S3**). In addition, some *Hyphomonas* sp. and *Undibacterium* sp. strains were isolated but they were not exclusive for this method. Experiments were performed twice in a 6-month interval (DTE1 and DTE2), and *Limnobacter* was, in both experiments, the most frequently isolated taxon. The ratio of cultivable to total bacteria (estimated culturability) in the experiment varied from 44 to 68%, with different culturability dependent on the type of bacterial growth medium applied. DTE statistics and the Poisson calculations can be found in **Table 1**.

**Table 1:** Estimation of the ratio of cultivable to total bacteria in the dilution-to-extinction cultivation experiments based on a Poisson distribution: n_cult_ = ln(1/p_neg_)^∗^w.

Experiment	Type of inoculation	Bacterial growth medium	# Bacterial cells inoculated (n_total_)	# Inoculated wells (w)	# Negative wells (P_neg_)	Theoretical # of cultivable cells (n_cult_)	Estimated culturability (n_cult_/n_total_)
DTE1	P	ECM 2W	52	104	74	35.39	68%
DTE1	P	ECM 7W	52	104	83	23.46	45%
DTE1	M	ECM 7W	48	96	73	26.29	55%
DTE1	M	ECM 2W	48	96	77	21.17	44%
DTE1	M	LNHM	52	104	81	25.99	50%
DTE1	M	1:20 R2A	52	104	79	28.59	55%
DTE2	M	1:20 R2A	140	280	201	92.82	66%

*P = protoplast digest product; M = low salinity algal growth medium; DTE1 = March 2016; DTE2 = September 2016; ECM = spent low salinity algal growth medium from 2 weeks (2W) or 7 weeks (7W) old cultures*.

#### Isolation of Bacteria from Algae with Reduced Flora

##### Antibiotic-treated algae

The 16S rRNA gene sequences from 80 isolates revealed 27 unique strains, 16 of which were obtained only with this cultivation method. *Bosea* was the most abundant (44% of isolates) followed by *Halomonas* with 38%. Most others were only isolated once or twice. Unique strains isolated with this method were *Sphingomonas* sp. (strains 1, 3), *Bacillus* sp. (strains 1, 3–5, 7), *Nocardioides* sp.*, Microcella* sp.*, Moraxella* sp. (strains 1, 2), *Pantoea* sp.*, Rhizobium* sp., two unclassified members of the *Flavobacteriaceae*, and *Oceanicaulis* sp. These were all (except *Microcella*) isolated from algal tissue that was exposed to 10 different antibiotics (**Supplementary Table S2**). The cultivation of bacteria from antibiotic-treated algae was performed twice within a 1-year interval and in both experiments *Bosea* was the most frequently isolated taxon.

##### Size-fractionation

The 16S rRNA gene sequences of the 43 isolates from this experiment cover 14 unique strains. Three of them were uniquely found using this method: *Bacillus* strain 6 (one isolate), *Limnobacter* strain 2, (two isolates), and *Stenotrophomonas* sp. (one isolate). Among the other strains cultivated, the most abundant one was *Limnobacter* strain 1 (40% of isolates), followed by *Imperialibacter* sp. (16% of isolates).

### Estimating the Proportion of Cultivable Bacteria in *Ectocarpus* Cultures

**FIGURE 3:**
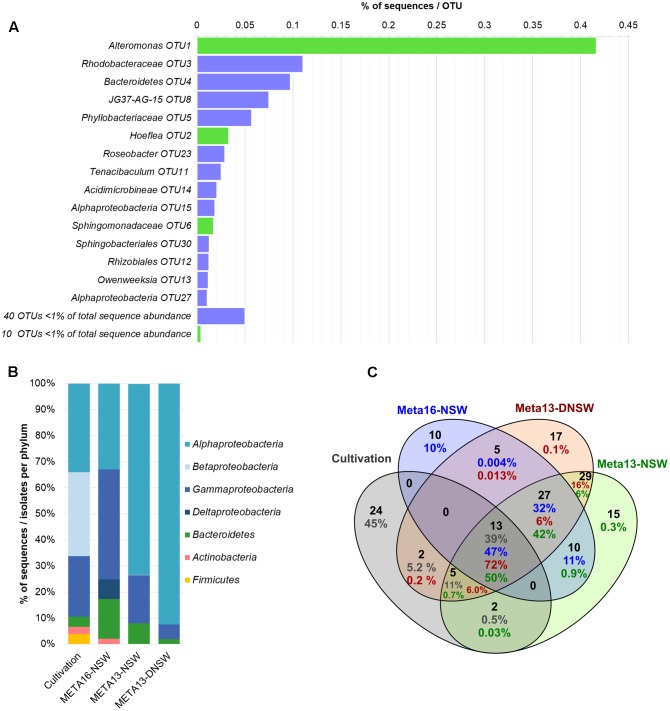
Overview of metabarcoding data and comparison with cultivable isolates. **(A)** Shows the distribution of OTUs in the metabarcoding experiment (META16-NSW). OTUs with >1% of total sequence abundance are displayed separately: bars in green display OTUs that correspond to cultivable strains obtained in this study, while purple bars correspond to OTUs that were not cultivated; OTUs with <1% of total sequence abundance are combined and the sum of sequences is displayed. **(B)** Shows the distribution of 16S rRNA gene metabarcoding sequences per phylum compared to data obtained from the cultivation study. **(C)** Shows a Venn-diagram of the OTUs that are shared between the 2 metabarcoding datasets from 2013 to 2016 and the cultivable isolates. Numbers in blue correspond to META16-NSW, numbers in red correspond to META2013-NSW, numbers in green to META2013-DNSW, and numbers in gray correspond to the proportion of sequences for cultivable isolates.

16S metabarcoding experiments were carried out with the same algal culture also used for the isolation of bacteria and the libraries were used as a reference for the cultivated and non-cultivated microbiome as a whole. After cleaning and filtering of the data, the sequences were clustered into 48 OTUs. The most abundant OTU belonged to the genus *Alteromonas* (OTU1) and accounted for 41.6% of the reads, which makes *Gammaproteobacteria* the most abundant class (42.3%). Other abundant OTUs corresponded to an unclassified *Rhodobacteraceae* (OTU3, 11%) and an unclassified *Bacteroidetes* (OTU4, 10%). Together these three OTUs correspond to 62% of all sequences (**Figure 3A**). *Alphaproteobacteria* make up 32.8% of the sequences and *Bacteroidetes* 15.3% (**Figure 3B**). Other phyla identified are *Actinobacteria* (2.1%) and *Deltaproteobacteria* (7.5%). Of the 48 OTUs, 10 corresponded to strains cultivated in our experiments. These 10 OTUs accounted for 47% of the reads in the metabarcoding data. Purely based on absence/presence of OTUs the culturability was 21%. Three additional cultivable strains corresponded to OTUs with sequence abundance below the threshold (*n* ≤ 5, *Staphylococcus, Hyphomonas, Oceanicaulis;* **Figure 3**). Furthermore, taking into account all 16S rRNA gene libraries and rare reads, 22 of the 46 cultivable strains were detected. Among the 24 undetected strains that were not found in any of the barcoding libraries, 11 were isolated exclusively from DNSW and 8 exclusively from NSW. In the same vein, 11 strains were cultured exclusively from algal medium, and 7 only from algal tissue (**Supplementary Table S4**).

The 16S rRNA gene metabarcoding data obtained in this study (META2016-NSW) differed strongly from that obtained 3 years earlier (META2013-NSW) from the same *Ectocarpus* strain. Several OTUs that were present in the 2013 samples were no longer present in 2016 or declined in abundance below the detection limit. However, there were still 50 OTUs (corresponding to 90% of the sequences) shared between the 2013 and 2016 samples. The most abundant OTU in 2013 belonged to the genus *Hoeflea* (29% of reads) while the most abundant OTU in 2016 (*Alteromonas* sp.; 42% of reads) accounted for only 2.4% of the reads in 2013.

## Discussion

### Global Taxonomic Distribution of Cultivable Bacteria

The main aim of this study was to establish a diverse collection of cultivable *Ectocarpus*-associated bacteria that can be used to perform functional studies of brown macroalgal–bacterial interactions in this model organism. We applied different cultivation strategies to facilitate the growth of less abundant or slow-growing bacteria and thus increased the variety of cultivable bacteria.

Among the cultivable taxa frequently found on brown macroalgal surfaces (such as *Laminaria*, *Saccharina*, *Fucus, Ascophyllum*) are *Proteobacteria*, *Firmicutes*, *Bacteroidetes*, and *Actinobacteria*, where the latter three phyla are generally less abundant (Ivanova et al., 2002; Wang et al., 2008; Wiese et al., 2009; Salaün et al., 2010; Dong et al., 2012; Goecke et al., 2013a,b; Hollants et al., 2013; Martin et al., 2015). Two cultivation studies in *Ectocarpus* species showed the presence of *Gammaproteobacteria*, *Actinobacteria*, and *Flavobacteria* (Kong and Kwong-yu, 1979; Tapia et al., 2016). The results of our study, with *Proteobacteria* being most abundant followed by *Bacteroidetes*, *Firmicutes*, and *Actinobacteria*, largely agree with these findings, except that *Alphaproteobacteria* were the most abundant proteobacteria in this study (**Figure 2** and **Supplementary Table S3**), compared to *Gammaproteobacteria* in the previous studies.

The three dominant genera obtained were *Limnobacter*, *Bosea*, and *Halomonas* (**Figure 2** and **Supplementary Table S3**). *Limnobacter* sp. are oligotrophic freshwater sulfur-oxidizing bacteria (Spring et al., 2001) that occur naturally in aquatic environments (Lu et al., 2011; Zhang et al., 2014) and drinking water reservoirs (Wu et al., 2014) and are generally considered rare in marine settings (Staufenberger et al., 2008; Wiese et al., 2009). The majority of the isolation experiments in this study were indeed carried out with low-salinity culture media, and the algal source also came from fresh water (West and Kraft, 1996), providing two possible explanations for the presence of *Limnobacter* in our experiments. As these experiments were always complemented with negative controls (i.e., non-inoculated bacterial growth media), a contamination with *Limnobacter* from the water source used to prepare the bacterial growth media is unlikely.

Members of the genus *Bosea* are known to be (multi)drug-resistant (Falcone-Dias et al., 2012; Zothanpuia et al., 2016). The results from our study agree with these observations since 92% of the *Bosea* isolates came from antibiotic-treated algae (**Figure 2** and **Supplementary Table S3**). In addition, several other strains were uniquely isolated from antibiotic-treated algal tissue suggesting that part of the algae-associated microbiome is (multi)drug-resistant, or otherwise protected by the algal cell wall/inside the cell, where drug concentrations may be too low to be effective. In addition, some antibiotics employed in this study, such as chloramphenicol and erythromycin, may have had only temporal bacteriostatic effects.

The genus *Halomonas* comprises cultivable isolates from various saline environments (Eilers et al., 2000; Donachie et al., 2004; Arahal and Ventosa, 2006; Poli et al., 2009), including microalgal (Keshtacher-Liebson et al., 1995; Croft et al., 2005; Baggesen et al., 2014) and macroalgal surfaces (Ivanova et al., 2002; Wang et al., 2008; Hollants et al., 2013; Tapia et al., 2016). *Halomonas*-algae associations are potentially beneficial for the alga, since the bacteria may provide vitamins (Croft et al., 2005), release siderophores (Keshtacher-Liebson et al., 1995; Baggesen et al., 2014), or excrete morphogenetic compounds (Spoerner et al., 2012; Tapia et al., 2016) that are essential for algal growth. Symbiotic associations with algae may be linked to the capacity of *Halomonas* to degrade algal excreted polysaccharides and/or the presence of alginate lyases (Wong et al., 2000; Tang et al., 2008; Wang et al., 2008; Goecke et al., 2012) and indeed, bacterial cells can be closely attached to algal cell walls (Croft et al., 2005; Tapia et al., 2016). In this study, *Halomonas* was the most abundant isolate obtained with direct plating techniques without pretreatment (DP1 and DP2). More isolates were derived from tissue/protoplasts compared to algal growth medium (*p* = 1.92E-13), suggesting a close association between *Ectocarpus* and the *Halomonas* sp.

In summary, each cultivation strategy resulted in the cultivation of unique strains that were not cultivated with any of the other methods. For example, the application of antibiotics to eliminate *Halomonas* sp. reduced the competitive pressure between antibiotic resistant bacteria and led to the cultivation of 16 additional bacterial strains. Similar observations have been made in sponges (Sipkema et al., 2011; Lavy et al., 2014), lichens (Parrot et al., 2015), and tap water (Vaz-Moreira et al., 2013). Interestingly, direct plating without pretreatment, although dominated by *Halomonas* sp., also resulted in the isolation of seven unique strains.

### The Cultivated vs. Uncultivated Microbiome

Marine pelagic bacteria often have complex growth and nutrient requirements (Stewart, 2012; Zengler, 2013). In addition, they are generally considered to be oligotrophs, since they inhabit a nutrient-poor environment and grow only very slowly, which might also compromise the cultivation process (Amann et al., 1995; Keller and Zengler, 2004; Zengler, 2013). Hence, a large part of the marine environmental microbiome has been considered non-cultivable using standard cultivation techniques (Amann et al., 1995; Morris et al., 2002; Giovannoni and Stingl, 2005).

Here, we aimed to cultivate bacteria that were associated with the algae/algal cell-walls, a carbohydrate-rich environment due to the accumulation of algal (poly)saccharides, i.e., alginates, fucans, and mannitol (Michel et al., 2010; Popper et al., 2011). The divergence in community structure between pelagic and algae-associated microbiomes is well-established (Kong and Kwong-yu, 1979; Staufenberger et al., 2008; Bengtsson et al., 2010; Burke et al., 2011; Wahl et al., 2012; Goecke et al., 2013b; Mancuso et al., 2016), and several algae-associated bacteria are able to digest/decompose algal cell material (Rieper-Kirchner, 1989; Goecke et al., 2013b; Groisillier et al., 2015; Martin et al., 2015), e.g., via the production of alginate lyases (Sawabe et al., 1997; Dong et al., 2012) and glycoside hydrolases/fucanases (Ficko-Blean et al., 2015). It is thus possible that algae-associated bacteria, contrary to pelagic bacteria, are well-adapted to grow on the laboratory cultivation media provided, resulting in relatively high numbers of cultivable bacteria.

Our data support this hypothesis since culturability was between 44 and 68% based on the dilution-to-extinction experiments (**Table 1**). For pelagic studies, culturability is usually below 15%, with some observation going as low as 0.05% (Connon and Giovannoni, 2002; Page et al., 2004; Stingl et al., 2007, 2008; Yang et al., 2016). In the same vein, dilution-to-extinction cultivation studies on pelagic bacteria generally apply between 1 and 25 bacterial cells/well as inoculum (Connon and Giovannoni, 2002; Stingl et al., 2007). In our study, however, concentrations as low as 0.5 cells/well were required to obtain pure cultures, further demonstrating that a relatively large part of the algae-associated microbiome is cultivable compared to pelagic bacteria.

Culturability was also assessed by comparing the distribution and abundance of taxa obtained in the cultivation study with taxa inferred from16S rRNA gene libraries. In a previous study on *Ectocarpus*, this type of comparison demonstrated an overall ratio of culturability of 11% based on the presence/absence of OTUs (Tapia et al., 2016). In the present study, this number was further increased with 21% of the OTUs and 47% of all 16S rRNA sequences corresponding to cultivable strains (**Figure 3C**).

To our knowledge, this is the first study to apply dilution-to-extinction cultivation to macroalgae-associated bacteria, and the standardized cultivation method (Connon and Giovannoni, 2002) was amended by adding the algal metabolites alginate and/or mannitol to the culture media. We assume that it was, therefore, well-adapted to the metabolic needs of the majority of *Ectocarpus*-associated bacteria, and indeed several bacteria known to be potential cell-wall digesters have representatives in our culture collection, e.g., *Alteromonas* (Sawabe et al., 1997), *Flavobacteria* (Groisillier et al., 2015), *Maribacter* (Martin et al., 2015), *Erythrobacter* (Goecke et al., 2013b), and *Halomonas* (Wong et al., 2000). Together these results validate the combination of cultivation approaches chosen to increase culturability in our system.

### Cultivable Bacteria Not Detected by Metabarcoding

Of the 46 unique strains that were isolated in this study, 16 were isolated at least once from algal tissue grown in NSW, and could thus be directly compared to META2016-NSW metabarcoding data set generated in this study. Seven of them (44%) were represented in this gene library. To be able to compare also strains isolated only from low salinities with metabarcoding data, we included two further data sets obtained for the same strain in 2013. All data sets taken together, 22 of the 46 (48%) strains were found at least in one of the libraries, while 24 were undetectable or below the detection limit. Whether a strain was isolated directly from algal tissue or from the algal culture medium did not have a strong impact on these numbers (**Supplementary Table S4**).

There are several hypotheses to explain this observation. First, methodological flaws or biases including the inadequacy to extract DNA from certain bacterial cells due to species-specific characteristics (e.g., gram-positive are generally more difficult to extract than gram-negative cells), primers specificity, or PCR conditions (Suzuki and Giovannoni, 1996; Donachie et al., 2004, 2007). This may explain biases but, is unlikely to account for the complete absence of a taxon, because all cultivable taxa were detectable with standard primers and extraction methods in our cultures. A second explanation is that some “rare” microbes may be laboratory- or human-derived contaminants, e.g., *Staphylococcus*, sp., and *Bacillus* sp. All measures to avoid bacterial contamination of our algal/bacterial samples were taken and the controls were included in all cultivation experiments and generally negative for growth. Nevertheless, it is plausible that some of these “rare” bacteria were acquired during the monthly transfers of the algal cultures or during the bacteria cultivation procedures and growth of these bacteria might have been facilitated by the experimental treatments that were applied.

A third explanation is that the sequencing depth or the number of time points examined (one for DNSW two for NSW) may have been too low to identify members of the microbiome that are “rare” (Zilber-Rosenberg and Rosenberg, 2008; Skopina et al., 2016). Bacteria might be present in low abundance in the natural environment but they can amplify rapidly under specific environmental conditions (Epstein, 2009; Buerger et al., 2012; Lindh et al., 2015). Rare bacteria might thus serve as a “seed bank” (Pedrós-Alió, 2012) that contributes to the microbial richness and may form the basis for temporal instability of the microbiome (Sogin et al., 2006; Shade and Gilbert, 2015; Jousset et al., 2017). Recently, it has been suggested that in particular marine macro-organisms, and possibly *Ectocarpus* as well, might serve as incubators for rare bacteria (Troussellier et al., 2017), since surface-associated microbiomes generally exhibit higher OTU diversity and harbor many rare OTUs compared to the surrounding seawater. In this scenario, the removal of competing microbes via the antibiotic treatments and our other measures to reduce competition during our cultivation experiments, have probably allowed them to increase in abundance. This explanation is supported by the variability of the microbiome observed in this study compared with the previous study of the same strain under the same conditions (Dittami et al., 2016), and by the fact that 4 of 46 cultured OTUs were found only among the rare (*n* ≤ 5) reads in the available barcoding data.

Similar observations of cultivable isolates not being detected in corresponding gene libraries have been made in human stool samples (Lagier et al., 2012), sponges (Sipkema et al., 2011; Esteves et al., 2016), seawater (Eilers et al., 2000), and soil (Shade et al., 2012); more examples are discussed by Donachie et al. (2007). We put forward the hypothesis that, in analogy to “uncultivable” microbes that become cultivable by improving cultivation conditions, at least part of the undetected strains may, therefore, become “barcodable” merely by significantly increasing sequencing depths (Pedrós-Alió, 2012) and/or the temporal resolution of the study.

### Perspectives: (Meta)genome-Guided Cultivation and Inference of Metabolic Networks

In this study, we show that a remarkably high number of bacterial cells (∼50%) associated with *Ectocarpus* was cultivable using a range of cultivation techniques. Each cultivation strategy resulted in another dominant genus or weed-species (*Bosea* for antibiotic-treated algae, *Limnobacter* for dilution-to-extinction cultivation) and each strategy also led to the cultivation of unique isolates that were not found with any other cultivation method. Our results thus emphasize the need to use samples from different environmental/abiotic conditions to obtain rare taxa and thus increase the overall cultivable diversity. To further improve these numbers, a metagenomics approach may be used to predict the specific cultivation requirements of yet uncultured taxa (Garza and Dutilh, 2015). One successful example of this approach is the cultivation of members of the SAR11 clade, for which genomic analysis revealed their requirement for exogenous reduced sulfur (Tripp et al., 2008). Such metagenomic analyses of the *Ectocarpus* holobiont are currently ongoing.

Regarding the cultivable isolates, genomic data currently in preparation for several strains may be used to predict their metabolic capacities and to generate hypotheses on how they may complement the metabolism of the alga (Dittami et al., 2014). Because the bacteria are cultivable it will be possible to experimentally verify the hypothesis generated using this approach. Sixty-two bacterial isolates and 12 artificial bacterial communities have already been experimentally tested in preliminary algal–bacterial co-culture experiments. They showed interactions ranging from weak beneficial effects on survival of *E*. *subulatus* in diluted natural seawater (29 isolates, 15 unique strains; three communities) to growth-inhibition (data not shown). These strains may serve as the first candidates to study the role of algal–bacterial interactions under abiotic stress.

The present bacterial culture collection constitutes a valuable tool to study the *Ectocarpus* holobiont *in vitro* and complements the genomic tools available for the model *Ectocarpus*. Together, they can be used to address fundamental questions regarding the functions of brown macroalgal holobionts during exposure to abiotic stressors, for instance during the acclimation to low salinity in *E. subulatus*.

## Author Contributions

All authors conceived the study. HK, CJ, and SD carried out experiments. HK wrote the manuscript with help from SD, CB, and CJ. All authors approved of the final manuscript.

## Conflict of Interest Statement

The authors declare that the research was conducted in the absence of any commercial or financial relationships that could be construed as a potential conflict of interest.

## Footnotes

1 https://blast.ncbi.nlm.nih.gov/Blast.cgi

2 http://mafft.cbrc.jp/alignment/software

3 https://www.bioinformatics.babraham.ac.uk/projects/fastqc/

4 http://hannonlab.cshl.edu/fastx_toolkit/index.html

5 https://www.mothur.org/wiki/MiSeq_SOP

6 http://itol.embl.de, version 3.5.3

7 http://stat.sys.i.kyoto-u.ac.jp/prog/pvclust/

## Supplementary Material

TABLE S1 Bacterial growth media recipes for LB, R2A, Zobell, PYG, EC-based, and LNHM.

TABLE S2 Antibiotics used to reduce the abundance of *Halomonas* sp.

TABLE S3 Number of bacterial isolates obtained per experimental treatment.

TABLE S4 Number of cultivable bacterial strains found in metabarcoding experiments depending on sample origin and sequencing library.
